# Impact of an acute geriatric outreach service to residential aged care facilities on hospital admissions

**DOI:** 10.1002/agm2.12176

**Published:** 2021-09-05

**Authors:** Jun Dai, Frank Liu, Deni Irwanto, Manoj Kumar, Nabaraj Tiwari, Jack Chen, Yinghua Xu, Matthew Smith, Daniel KY Chan

**Affiliations:** ^1^ Aged Care and Rehabilitation Department Bankstown‐Lidcombe Hospital Bankstown NSW Australia; ^2^ Ingham Institute & Simpson Centre for Health Services Research SWS Clinical School/UNSW Sydney NSW Australia; ^3^ Faculty of Medicine University of New South Wales Kensington NSW Australia

**Keywords:** Geriatric, health service evaluation, health services for the aged, hospital avoidance, hospital in the nursing home, residential facilities, hospital avoidance

## Abstract

**Introduction:**

Residential aged care facility (RACF) residents frequently present to the emergency department (ED) and are often admitted to hospital. Some presentations and admissions may be avoidable. In 2013, Bankstown‐Lidcombe Hospital introduced a subacute geriatric outreach service (SGOS), which had little impact on reducing ED presentations. In 2015, Bankstown‐Lidcombe Hospital introduced an acute geriatric outreach service (AGOS), a geriatrician‐led team that assesses and treats acutely unwell patients in RACFs. We aim to determine whether the AGOS reduces the risk of hospital admission for RACF residents.

**Methods:**

Hospital admissions data from 2010 to 2019 were used to conduct an interrupted time series (ITS) analysis. AGOS activity data were also summarized.

**Results:**

The average number of admissions from RACF per month declined from 42.8 during the SGOS period to 27.1 during the AGOS period. The difference of 15.7 admissions from RACF per month was statistically significant (95% CI 12.1–19.2; *P* < .001). After the introduction of the AGOS, the risk of admission to our geriatric department from RACFs was reduced by 36.1% (incidence rate ratio =0.64; 95% CI: 0.58–0.71; *P* < .001) compared to the SGOS period, adjusting for seasonality.

**Discussion:**

The AGOS probably reduced the risk of hospital admission for RACF residents.

## Graphical abstract

Monthly number of geriatric admissions to Bankstown‐Lidcombe Hospital from residential aged care facilities (2010–2019)

1. Subacute Geriatric Outreach Service introduced
2. Acute Geriatric Outreach Service introduced
3. 36% reduction in risk of hospitalization from RACF

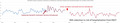

## 1 INTRODUCTION

Australians aged 65 and older are projected to rise from 15% in 2016 to 19% by 2030.^1^ Over 170, 000 adults aged 65 and older live in residential aged care facilities.^2^ People living in residential aged care facilities (RACFs) are often frail and have medically complex care needs. The average age of RACF residents is 84.5 years. Fifty percent of residents have dementia, 26% have a mental illness but no dementia, and 22% neither have dementia or a mental illness. Twenty‐nine percent of male and 18% of female residents have had a stroke, head injury, or acquired brain injury. Fifty‐four percent of residents have a musculoskeletal disorder, 18% have heart disease, 10% have another neurological disorder, and 7% have cancer.^3^

RACF residents are at greater risk of emergency department (ED) representations and hospital readmissions.^4^ They present to EDs at rates of 0.1–1.5 transfers per RACF bed/year.^5^ Up to 60% of these presentations are subsequently admitted to hospital.^6^, ^7^ These ED presentations and hospital admissions can potentially be avoided by treating patients safely in the RACF. This can reduce incident delirium from entering a new environment, nosocomial infections, medication errors, pressure injuries, falls, and resource utilization.^8^, ^9^, ^10^

In 2013, Bankstown‐Lidcombe Hospital introduced a service improvement initiative supported by the hospital called the subacute geriatric outreach service (SGOS), where a geriatrician would visit RACFs to manage behavioral and psychological symptoms of dementia (BPSD) and follow up discharged patients who were admitted recently for acute medical conditions. This service had little impact on reducing ED presentations, so in 2015, Bankstown‐Lidcombe Hospital introduced another service improvement initiative supported by the hospital called the acute geriatric outreach service (AGOS) that received referrals of acutely unwell patients from RACFs in the Bankstown catchment area. The AGOS geriatricians and nurse triage these referrals and visit RACFs to assess and manage patients there with “hospital in the home” interventions if possible.

We have previously shown that there was a decrease in the number of ED presentations from RACFs after the introduction of the AGOS.^11^ However, data available at the time (1 June 2013 to 30 April 2017) could only demonstrate a trend toward a reduction in geriatric department hospital admissions from RACFs, likely due to lack of power from available sample size.

### 1.1 Aims and hypothesis

We aim to determine if there is any change in the number of geriatric department hospital admissions from RACF after introduction of the AGOS by analyzing a larger data set (1 January 2010 to 31 December 2019). We hypothesize that this larger data set will yield a statistically significant reduction.

## 2 METHODS

### 2.1 Study design

We conducted an ITS analysis. There were three study periods.

The preintervention period is defined as the 41 months from 1 January 2010 to 31 May 2013 inclusive. During this time, Bankstown‐Lidcombe Hospital did not provide any outreach services for RACFs. General practitioners provided nonurgent care to RACF residents, while acutely unwell patients were referred to the emergency department.

The SGOS period is defined as the 19 months from 1 June 2013 to 31 December 2014 inclusive. In June 2013, Bankstown‐Lidcombe Hospital introduced a subacute geriatric outreach service (SGOS). This service consisted of a 0.6 full time equivalent (FTE) geriatrician that visited RACF to manage subacute problems such as behavioral and psychological symptoms of dementia (BPSD), follow up reviews after discharge from hospital, and symptom management for palliative patients. A geriatrician working three days a week saw approximately 400 referrals annually, equating to 8 patient encounters per week. Acutely unwell patients were still referred to the emergency department during this period. About 80% of RACF visits were for BPSD management.

In May 2015, Bankstown‐Lidcombe Hospital introduced an AGOS. It is comprised of one FTE geriatrician, one FTE aged care nurse, and one FTE geriatric trainee. It receives referrals of acutely unwell patients from 17 RACFs in the Bankstown catchment area. The geriatrician and aged care nurse triage these referrals and visit RACFs to assess and manage patients there if possible. The service delivers “hospital in the home” interventions such as cannulation, intravenous drugs (antibiotics, furosemide), and subcutaneous fluids. Other interventions provided include symptom management, difficult urinary catheterizations, advance care planning, and medication reviews. Details of medical conditions treated are listed in Table 1. The service relies on private pathology and radiology providers for investigations. It works collaboratively with private wound nurse practitioners and community nursing services. Hyperacute problems such as stroke and acute coronary syndromes are excluded from this service and are referred to the ED, since private pathology and radiology providers have a slower turnaround time for investigations results: investigations are often performed the following day, with results available the day after. This means the AGOS often initiate empirical therapy for common conditions based on clinical suspicion. The aged care nurse collects data on referrals and treatment outcomes to maintain a quality database.

The AGOS period is defined as the 4 years from 1 January 2016 to 31 December 2019 inclusive. During this time, the AGOS was well established and operational. 2015 was treated as a transitional period to allow RACFs time to adjust their referral patterns after being introduced to a new service model. Data from 2015 were omitted from the final analysis.

**TABLE 1:** Acute Geriatric Outreach case mix (top 10 conditions treated in 2018)

Respiratory infections	106
Urinary infections	73
Dehydration	35
Skin infections	26
Urinary catheter issues	20
Exacerbation of heart failure	17
Exacerbation of COPD	10
Abdominal pain	8
Other infections	6
BPSD	5

### 2.2 Data Sources/Collection

We extracted variables from the AGOS database such as the demographics and acuity of a referral, conditions treated, and treatment outcome. We summarize this data using descriptive statistics to give a snapshot of service activity and case mix.

We obtained admissions data from our hospital's clinical information unit. They generated a list of all patients admitted to our geriatrics department from 1 January 2010 to 31 December 2019. After excluding the data for 2015 (transitional period), there were 24,331 hospital admissions to our geriatric department during the study period.

### 2.3 Population/Sample Size

There are currently 17 RACFs in the Bankstown catchment area. Twelve of these were operational during the whole study period so they were included in the analysis, while the other five RACFs were excluded because they opened midway into the study (i.e. time varying confounders). The total number of beds in the 12 study RACFs was 1421 in 2012. This number grew over time to 1491 in 2019.

### 2.4 Ethics

Ethics approval was granted by South Western Sydney Local Health District Human Research Ethics Committee (2019/ETH11733). A waiver of consent was sought as it was impractical to obtain consent for two reasons: a number of individuals have passed away since the health information was originally collected, and the difficulty of contacting individuals directly when there is no existing or continual relationship with them.

### 2.5 Statistical Methods

We used independent *t* test to compare the means of each study period's monthly total number of admissions from RACFs, taking outliers into account. We performed negative binomial regression modeling for hospital admissions, adjusting for seasonality. We used STATA 16 (StataCorp) for statistical analysis.

## 3 RESULTS

### 3.1 Summary of the AGOS Data

The AGOS provides 546 to 571 care episodes annually, equating to 45.5 to 47.6 care episodes provided per month. A care episode consists of an initial individual patient's assessment plus any additional follow‐up visits of the same patient until they are discharged from our care. The total number of RACF visits per year is larger and range from 971 to 1292. The majority of referrals were triaged as acute (62.1%–73.4%). The three commonest acute conditions treated were pneumonia, urinary tract infections, and dehydration. Adverse events included 2–12 incidents per year of hospitalization after initial treatment in the nursing home.

### 3.2 Impact on hospital admissions from the 12 RACFs

Over the 41 preintervention months (1 January 2010 to 31 May 2013), there were 9834 geriatric department inpatient admissions, of which 1496 (15.2%) were from a RACF. Over the 19 months of the SGOS period (1 June 2013 to 31 December 2014), there were 4746 geriatric department inpatient admissions, of which 812 (17.1%) were from a RACF. Over the 4 years of the AGOS period (2016–2019), there were 9751 geriatric department inpatient admissions, of which 1302 (13.4%) were from a RACF.

Independent *t* test showed that following the establishment of the SGOS, there was a higher number of monthly admissions from RACF (42.8 ± 7.5) compared to monthly admissions prior to its establishment (36.5 ± 6.0). The difference of 6.3 admissions from RACF per month was statistically significant (95% CI 2.7–9.9; *P* <.001). The number of monthly geriatrics admissions for non‐RACF patients were similar for both periods (203.5 ± 26.6 monthly admissions during the preintervention period versus 206.0 ± 32.2 monthly admissions during the SGOS period, t(58) = −0.32, *P* =.75). The number of monthly geriatrics admissions overall, both RACF and non‐RACF patients, were similar for both periods (239.9 ± 29.0 monthly admissions during the preintervention period versus 248.8 ± 34.9 monthly admissions during the SGOS period, t(58) = −1.03, *P* =.31).

In contrast, following the establishment of the AGOS, there was a lower number of monthly admissions from RACF (27.1 ± 6.2) compared to the SGOS period (42.8 ± 7.5). The difference of 15.7 admissions from RACF per month was statistically significant (95% CI 12.1–19.2; *P* <.001). Negative binomial regression showed that during the AGOS period, risk of admission to our geriatric department was reduced by 36.1% compared to the SGOS period (incidence rate ratio =0.64; 95% CI: 0.58–0.71; *P* <.001), adjusting for seasonality.

The number of monthly geriatrics admissions for non‐RACF patients also decreased during this period (206.0 ± 32.2 monthly admissions during the SGOS period versus 176.0 ± 18.4 monthly admissions during the AGOS period, t(65) = −4.79, *P* <.001).

The number of monthly geriatrics admissions overall, including patients not from a RACF, decreased during this period (248.8 ± 34.9 monthly admissions during the SGOS period versus 203.1 ± 20.8 monthly admissions during the AGOS period, t(65) = 6.60, *P* <.001).

**FIGURE 1:**
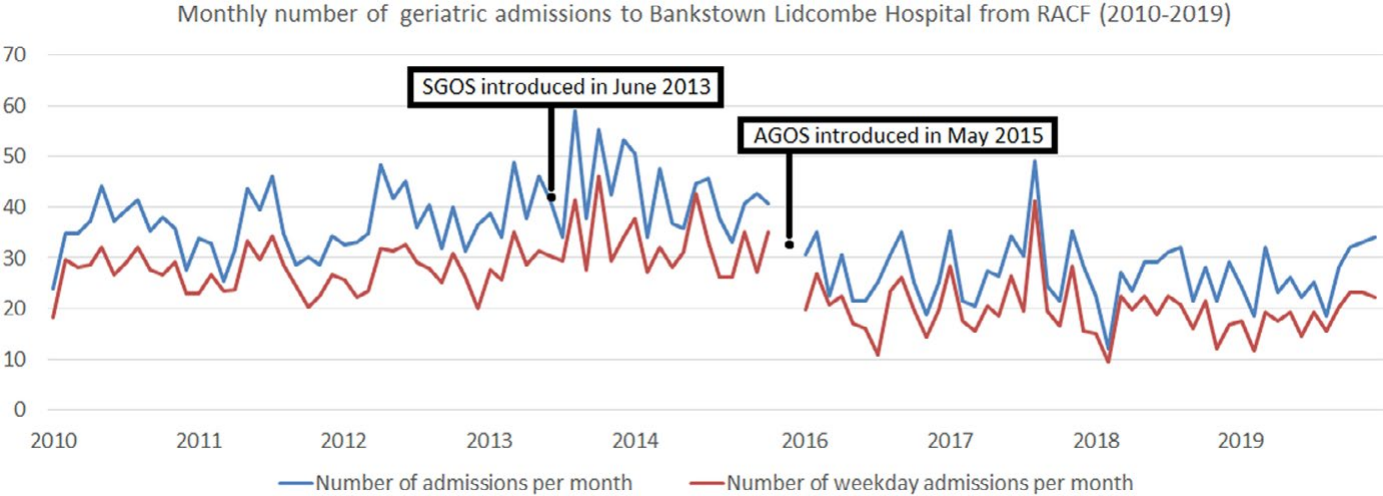
Monthly number of geriatric admissions to Bankstown‐Lidcombe Hospital from RACF (2010–2019)

Since there was no coverage of our service on weekends, we performed another analysis that excluded weekend admissions from RACF. In this analysis, the independent *t* test also showed that following the establishment of the AGOS, there was a lower number of weekday admissions from RACF (19.76 ± 5.33) per month compared to the SGOS period (32.6 ± 5.8). The difference of 12.8 monthly admissions from RACF was statistically significant (95% CI 9.9–15.8; *P* <.001). Negative binomial regression showed that postintervention, risk of admission to our geriatric department was reduced by 39% (incidence rate ratio =0.61; 95% CI: 0.54–0.68; *P* <.001). There was no evidence of seasonality (Figure 1).

## 4 DISCUSSION

We demonstrated a 36.1% reduction in the risk of hospital admission for RACF residents after the introduction of the AGOS, adjusting for seasonality. Reduction in hospital admission risk was greater during weekdays (39%) as expected since there's no weekend service. The mean reduction of 12.8 weekday inpatient admissions from RACF per month is reflected by the corresponding number of acute referrals managed by the AGOS per month (28.25 to 34.92). Over this time, the 12 RACFs included in our study expanded their total bed number by 4.9% and received 59.2% more government funding.^12^

Previous feasibility studies of our acute service during the early days showed a 10.2% reduction in ED transfers from RACFs and an increased rate of discharge from ED for RACF residents (38.4% vs 52.5%, odds ratio=1.76, 95% CI 1.2–2.4, *P* <.001) compared to the SGOS period.^11^, ^13^ ITS analysis using unpublished data from those study periods found a statistically nonsignificant 26% reduction in the risk of admission from RACF. The statistically significant larger effect size in our current study could be explained by the fact that our service has matured over time, with a stronger referral base and a bigger sample size with adequate power.

Our findings are consistent with other RACF outreach services but the models vary. The other services are variably known as the “Geriatric Flying Squad,” “Residential In‐Reach,” “Hospital in the Nursing Home (HINH),” and “RACF Hospital Avoidance Service.”

The South Care Geriatric Flying Squad is a RACF outreach service in Sydney, Australia with similar referral criteria and case mix. Compared to our AGOS, they're better equipped with portable point‐of‐care testing, blood pathology lab, bladder scanner, and ECG. Jain et al. speculates that their service may have averted ED transfers for 90.3% of RACF referrals.^14^ However, their study differed from our previous and current study. They assume a referral they see would equate to a definite episode of ED avoidance (that is they do not have a control group), while we performed ITS analysis of ambulance and hospital data to demonstrate a reduction in the number of ED transfers and hospital admissions between pre‐ and postintervention periods. We believe our approach is more robust.

There are multiple Residential In‐Reach services in Victoria, Australia. The in‐reach team consists of a mix of nurse practitioners and geriatricians who visit RACFs to manage unwell residents. Some teams are nurse led while others are geriatrician led. In some centers, they collaborate with Hospital in the Home (HITH) and mobile X‐ray services. Retrospective audits show a reduction in ED presentations and representations, hospital admissions, and ED length of stay after introduction of these various services; however, their pre‐ and postintervention time periods are not as extensive as our current study.^15^, ^16^, ^17^

Dwyer et al. performed a qualitative evaluation of the Central Queensland Hospital's RACF hospital avoidance service. This service differs from our AGOS in that it is nurse practitioner led service. Medical governance remains with the patient's primary care provider, but nurse practitioners liaise with emergency physicians if the patient's primary care provider is unavailable. The nurse practitioners are able to deliver a range of timely health services within the RACF due to their advanced clinical skills and prescribing rights. However, their lack of the access to Medicare Benefits Schedule rebates restricts their scope of practice.^18^

Crilly et al. performed a matched cohort study of Gold Coast Hospital's Hospital in Nursing Home (HINH) and found the service reduced in‐hospital LOS. While this service allowed RACF residents to receive treatments such as intravenous antibiotics in their homes, study participants all presented to ED for initial medical assessment prior to being enrolled onto the HINH program.^19^, ^20^ In contrast, our AGOS and other Residential In‐Reach services visit RACFs to assess acute referrals to avoid ED transfer.

Our study has limitations. We only examined hospital admissions to our geriatric department. While some RACF patients would be admitted under surgery or other medical specialties, most admissions from RACF would be under geriatrics medicine, so this study would have captured most of the data. We did not examine admissions to other hospitals from the 12 RACFs. This would be relevant for RACFs located on the border of our LHD, since residents of those RACF might be transferred to other hospitals. However, most admissions to hospitals are locally based and the confounding effect would have affected both before and after periods of AGOS.

External factors may also have influenced the number of hospital admissions from RACF. In recent years, Australian health policy supported aging at home by providing more funding for home care packages. This may have resulted in low‐care patients staying in their own homes longer, shifting the RACF demographic to a frailer population who require high‐level care. This would increase the risk of hospitalizations from RACFs, causing our study to underestimate AGOS’s contribution in reducing hospital admissions from RACF.

Other health care service providers to RACFs include general practitioners, private geriatricians and psychogeriatricians, private nurse practitioners, palliative care outreach services, and dementia‐related support programs, e.g. Dementia Behavior Management Advisory Service (DBMAS). It is possible that increased activity from these other providers may have also contributed to a decline in the number of hospital admissions from RACF. A comparative ITS analysis, using admissions data from a neighboring local health district without a RACF outreach service for comparison, will better control for external confounders .

## 5 CONCLUSION

The results of our study support our hypothesis that the AGOS reduced hospital admissions. Future studies could better address external confounders by adopting a comparative interrupted time series design and expanding the AGOS database to include additional parameters.

## CONFLICT OF INTEREST

Nothing to declare.

## AUTHOR CONTRIBUTIONS

J. Dai contributed to ethics submission, study design, analysis, writing, and preparation of the manuscript; DKY Chan, study design, analysis, writing, and preparation of the manuscript; N. Tiwari, data collection; YH Xu, ethics submission; F. Liu, preparation of the manuscript; D. Irwanto, preparation of the manuscript; J. Chen, preparation of the manuscript; M. Smith, preparation of the manuscript. The author Daniel KY Chan is a member of Aging Medicine’s editorial board and excluded from the handling of this manuscript.
